# Transition to rilonacept monotherapy from oral therapies in patients with recurrent pericarditis

**DOI:** 10.1136/heartjnl-2022-321328

**Published:** 2022-10-31

**Authors:** Antonio Brucato, Alistair Wheeler, Sushil Allen Luis, Antonio Abbate, Paul C Cremer, Liangxing Zou, Antonella Insalaco, Martin Lewinter, Basil S Lewis, David Lin, Stephen Nicholls, Massimo Pancrazi, Allan L Klein, Massimo Imazio, John F Paolini

**Affiliations:** 1 Department of Biomedical and Clinical Sciences "Sacco", University of Milano, Milano, Italy; 2 Clinical Development, Kiniksa Pharmaceuticals Corp, Lexington, Massachusetts, USA; 3 Department of Cardiovascular Medicine, Mayo Clinic College of Medicine, Rochester, Minnesota, USA; 4 Division of Cardiology, Pauley Heart Center, Virginia Commonwealth University, Richmond, Virginia, USA; 5 Cardiovascular Medicine, Heart and Vascular Institute, Cleveland Clinic, Cleveland, Ohio, USA; 6 Division of Rheumatology, Ospedale Pediatrico Bambino Gesù, Roma, Lazio, Italy; 7 Cardiology Unit, University of Vermont Medical Center, Burlington, Vermont, USA; 8 Department of Cardiology, Lady Davies Carmel Medical Center, Haifa, Haifa, Israel; 9 Department of Cardiology, Minneapolis Heart Institute, Minneapolis, Minnesota, USA; 10 Monash Cardiovascular Research Centre, Victorian Heart Institute, Monash University, Clayton, Victoria, Australia; 11 Department of Internal Medicine, Fatebenefratelli Hospital, Milan, Italy; 12 Cardiothoracic Department, Santa Maria della Misericordia University Hospital, Udine, Friuli-Venezia Giulia, Italy

**Keywords:** pericarditis

## Abstract

**Objective:**

Polypharmacy management of recurrent pericarditis (RP) often involves long-term therapies, often with negative effects. Slow tapering of oral therapies is often required to avoid recurrence. A post hoc analysis of the phase III trial Rilonacept inHibition of interleukin-1 Alpha and beta for recurrent Pericarditis: a pivotal Symptomatology and Outcomes Study (RHAPSODY) evaluated investigator approaches to transitioning to IL-1 blockade monotherapy with rilonacept, which was hypothesised to allow accelerated withdrawal of common multidrug pericarditis regimens.

**Methods:**

RHAPSODY was a multicentre (Australia, Israel, Italy, USA), double-blind, placebo-controlled, randomised-withdrawal trial in adults and adolescents with RP. Investigators initiated rilonacept at the labelled dose level and discontinued oral pericarditis therapies during the 12-week run-in; randomised patients received study drug as monotherapy. Time to rilonacept monotherapy was quantified in patients receiving multidrug regimens at baseline who achieved rilonacept monotherapy during run-in.

**Results:**

In 86 enrolled patients, mean time to rilonacept monotherapy was 7.9 weeks, with no recurrences. Of these, 64% (n=55) entered on multidrug regimens: non-steroidal anti-inflammatory drugs (NSAIDs) plus colchicine (44% (24/55)), colchicine plus glucocorticoids (24% (13/55)), or NSAIDs, colchicine, plus glucocorticoids (33% (18/55)). Investigators transitioned patients receiving colchicine and glucocorticoids at baseline to rilonacept monotherapy without recurrence regardless of taper approach: sequential (n=14; median, 7.7 weeks) or concurrent (n=17; median, 8.0 weeks). Median time to rilonacept monotherapy was similar regardless of glucocorticoid dose and duration: ≤15 mg/day (n=21): 7.3 weeks; >15 mg/day (n=18): 8.0 weeks; long-term (≥28 days): 7.6 weeks.

**Conclusions:**

Rapid discontinuation of oral RP therapies while transitioning to rilonacept monotherapy was feasible without triggering pericarditis recurrence.

**Trial registration number:**

NCT03737110.

**WHAT IS ALREADY KNOWN ON THIS TOPIC**

- Management of recurrent pericarditis (RP) involves polypharmacy, including long-term glucocorticoid treatment, which is associated with adverse effects. Rapid tapering of glucocorticoids has been associated with increased risk of pericarditis recurrence, and so clinicians often slowly taper oral pericarditis therapies. The phase III trial Rilonacept inHibition of interleukin-1 Alpha and beta for recurrent Pericarditis: a pivotal Symptomatology and Outcomes study (RHAPSODY) demonstrated the efficacy and safety of rilonacept, an IL-1α and IL-1β cytokine trap, for RP.

**WHAT THIS STUDY ADDS**

- RHAPSODY provided a controlled environment to test the hypothesis that efficacious IL-1 blockade may render standard therapies redundant, challenging the traditional slow-tapering approach. Of 86 patients participating in the RHAPSODY run-in period, >60% were taking combination therapies. This post hoc analysis of data from the phase III trial RHAPSODY assessed how study investigators transitioned patients with RP who were receiving common multidrug regimens to rilonacept monotherapy. Investigators successfully transitioned all patients receiving non-steroidal anti-inflammatory drugs, colchicine, and glucocorticoids at baseline to rilonacept monotherapy without recurrence using either a sequential (median, 7.7 weeks) or concurrent (median, 8.0 weeks) approach. In addition, rilonacept therapy allowed for rapid discontinuation of oral RP therapies without triggering pericarditis recurrence regardless of dose or duration of glucocorticoid treatment.

**HOW THIS STUDY MIGHT AFFECT RESEARCH, PRACTICE OR POLICY**

- These findings provide insight for clinicians transitioning patients with RP to rilonacept monotherapy, affirming a strategy of rapid cessation of oral therapies. These findings suggest efficacious IL-1 blockade may render standard oral therapies redundant, supporting accelerated withdrawal.

## Introduction

Recurrent pericarditis (RP) is a debilitating disease with autoinflammation being one of the possible underlying pathogenic mechanisms. RP is characterised by repeated episodes of pericardial inflammation causing chest pain, ECG changes and pericardial effusion.^1–3^ Current management of RP includes treatment with non-steroidal anti-inflammatory drugs (NSAIDs), colchicine, systemic glucocorticoids; third-line medications include azathioprine and intravenous human immunoglobulins, alone and in various combinations.^1^ After the first episode, additional episodes occur in 15%–30% of patients, and management of RP often requires treatment lasting months to years.^1 4 5^ NSAIDs and colchicine are associated with gastrointestinal and renal side effects that limit use in some patients. Many patients require long-term use of glucocorticoids, which can cause serious adverse effects, including diabetes, hypertension, osteoporosis and adrenal insufficiency.^6–12^ A history of glucocorticoid treatment is an independent risk factor for pericarditis recurrence,^13^ and premature tapering of glucocorticoids may contribute to recurrence.^1^ For these reasons, guidelines recommend tapering glucocorticoids gradually, over several months.^1^ Considering the increased risk and seriousness of adverse events associated with long-term glucocorticoid treatment,^14 15^ a need exists for therapies that effectively prevent pericarditis recurrence while minimising glucocorticoid exposure.

The cytokine interleukin (IL) 1 is a key mediator in the inflammatory processes involved in RP.^8 16^ Rilonacept, an IL-1α and IL-1β cytokine trap, demonstrated efficacy and safety in patients with RP in the phase III Rilonacept inHibition of interleukin-1 Alpha and beta for recurrent Pericarditis: a pivotal Symptomatology and Outcomes Study (RHAPSODY).^17^ Rilonacept therapy rapidly resolved pericarditis episodes after initiation during the run-in period and significantly reduced the risk of pericarditis recurrence versus placebo during the placebo-controlled, randomised-withdrawal period.^17^ These data supported the US Food and Drug Administration approval of rilonacept as the first therapy indicated for RP.^18^ The non-specific anti-inflammatory mechanisms of standard oral therapies overlap the targeted immunomodulatory mechanism of rilonacept, and the randomised-withdrawal period was designed to compare rilonacept monotherapy with placebo. Investigators in RHAPSODY initiated rilonacept therapy by adding it to standard oral therapies and subsequently discontinuing standard oral therapies over 8 weeks.^17 19^ Tapering of oral therapies, including glucocorticoids, was designed to take place more rapidly than current clinical practice and guideline recommendations because the mechanism of action of rilonacept, that is, selective blockade of the IL-1 pathway, was hypothesised to be sufficient as monotherapy to control RP.^1 19^ This post hoc analysis provides empirical data describing how investigators managed NSAID, colchicine, and glucocorticoid therapies while transitioning patients to rilonacept monotherapy during the run-in period of RHAPSODY.

## Methods

### Study design

The study design and CONSORT diagram of RHAPSODY were reported previously.^20^ Briefly, this was a phase III, multicentre, double-blind, placebo-controlled, randomised-withdrawal trial conducted in Australia, Israel, Italy and the USA. Patients were screened for up to 4 weeks to determine trial eligibility and then entered a 12-week run-in period. Adult patients (≥18 years of age) received an initial loading dose of rilonacept 320 mg subcutaneously at baseline, followed by a 160 mg dose administered once weekly throughout the run-in. Adolescent patients (≥12 years to <18 years of age) received an initial loading dose of rilonacept 4.4 mg/kg subcutaneously at baseline, followed by a 2.2 mg/kg dose administered once weekly throughout the run-in.

The run-in period was followed by a randomised-withdrawal period, during which eligible patients were randomised to receive continued rilonacept or placebo. Once the prespecified number of adjudicated pericarditis recurrences had accrued, the randomised-withdrawal phase was closed, and eligible patients had the option to continue open-label rilonacept during a long-term extension.

**Figure 1:**
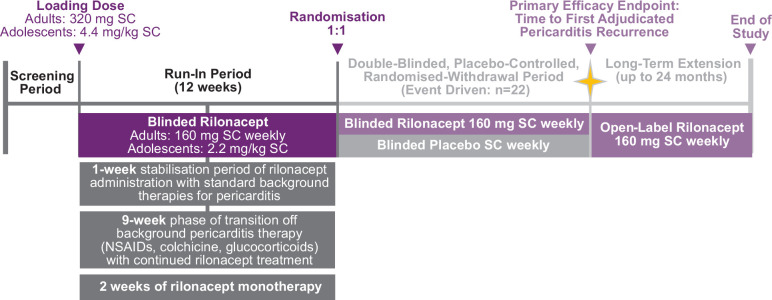
Study design of Rilonacept inHibition of interleukin-1 Alpha and beta for recurrent Pericarditis: a pivotal Symptomatology and Outcomes study (RHAPSODY) run-in period. NSAID, non-steroidal anti-inflammatory drug; SC, subcutaneous.

The goal of the run-in period was to prepare patients for randomisation to rilonacept or placebo at week 12. To accomplish this and to ensure clinical stability on rilonacept monotherapy at randomisation, attainment of rilonacept monotherapy was required by week 10. The run-in period included a 1-week stabilisation period of rilonacept administration with standard oral therapies (NSAIDs, colchicine, glucocorticoids), a 9-week phase of transition off oral therapies with continued rilonacept treatment, and 2 weeks of rilonacept monotherapy (figure 1). The study protocol did not stipulate specific timing or dosing guidance during the transition, but it did require that patients reach monotherapy rilonacept by week 10 and continue through week 12 to qualify for randomisation. The protocol recommended that once clinical stability had been achieved, investigators should begin with glucocorticoid tapering (from week 1 up to week 9), while tapering of NSAID and colchicine should begin later (weeks 4–10), unless earlier dose reduction was needed. The guidelines for background medication taper and discontinuation are shown in table 1.

**Table 1:** Proposed medication tapering schedule

Pericarditis medication	Starting dose (mg)	Week 1 (mg)	Week 2 (mg)	Week 3 (mg)	Week 4 (mg)	Week 5 (mg)	Week 6 (mg)	Week 7 (mg)	Week 8 (mg)	Week 10
Prednisone	60	30	20	15	10	7.5	5	2.5	1	Stop
	50	25	20	15	10	7.5	5	2.5	1	Stop
	40	20	15	15	10	7.5	5	2.5	1	Stop
	35	17.5	15	10	7.5	5	2.5	1	Stop	
	30	15	10	10	7.5	5	2.5	1	Stop	
	25	12.5	10	10	7.5	5	2.5	1	Stop	
	20	10	7.5	7.5	7.5	5	2.5	1	Stop	
	15	7.5	7.5	5	5	2.5	2.5	1	Stop	
	12.5	7.5	7.5	5	5	2.5	2.5	1	Stop	
	10	5	5	5	2.5	2.5	1	Stop		
	7.5	5	5	2.5	2.5	2.5	1	Stop		
	5	2.5	2.5	2.5	1	Stop				
	2.5	2	2	1	Stop					
Analgesics		Start taper or stop	Taper or stop	Taper or stop	Taper or stop	Taper or stop	Taper or stop	Taper or stop	Taper or stop	Stop
NSAIDs					Start taper	Taper or stop	Taper or stop	Taper or stop	Taper or stop	Stop
Colchicine					Start taper	Taper or stop	Taper or stop	Taper or stop	Taper or stop	Stop

NSAIDs, non-steroidal anti-inflammatory drugs.

### Patients

The study enrolled adult and adolescent (≥12 years of age) patients with a diagnosis of recurrent idiopathic or postcardiac injury pericarditis, defined as two or more recurrences after the first pericarditis episode. Eligible patients could be taking any combination of NSAIDs, colchicine or glucocorticoids. Eligible patients were required to have 1 day or more with pericarditis pain severity 4 or greater on an 11-point Numerical Rating Scale and C reactive protein level of at least 1.0 mg/dL within 7 days before first administration of rilonacept. Pericarditis recurrence required these scores for pericarditis signs/symptoms, whereas clinical response required a pericarditis pain severity score of 2 or less with a C reactive protein level ≤0.5 mg/dL.

### Analyses

This post hoc analysis evaluated path and time to rilonacept monotherapy in a group of patients who were receiving combination therapy with NSAIDs, colchicine, and/or glucocorticoids at baseline and who achieved rilonacept monotherapy during the run-in period. Of the 86 subjects that enrolled in the trial, 64% (n=55) entered on multidrug regimens. An ad hoc analysis was performed to assess the length of time necessary to achieve monotherapy. Patients were divided into three groups based on type of combination therapy: NSAID and colchicine double therapy; colchicine and glucocorticoid double therapy; and NSAID, colchicine and glucocorticoid triple therapy. Time to rilonacept monotherapy was also evaluated in patients with long-term (≥28 days) glucocorticoid treatment, with or without other pericarditis treatments, and in subgroups based on glucocorticoid dose (≤15 mg/day (median study dose) or >median study dose), annualised incidence (<2 episodes, 2–4 episodes, or >4 episodes) and disease duration (<1 year, 1–2 years, or >2 years). In patients receiving treatment with both colchicine and glucocorticoids, time to rilonacept monotherapy was assessed in subgroups stratified by taper approach. Those who completed tapering of glucocorticoid prior to beginning colchicine dose reduction/discontinuation were considered to have a ‘sequential’ taper. Those who began colchicine dose reduction/discontinuation during glucocorticoid taper were considered to have a ‘concurrent’ taper. Although sample size is small in this study, the mean was close to median for most continuous variables. In general, continuous variables are presented with descriptive mean and SD. In case mean deviates dramatically from median, median (Q1, Q3) is presented. For time to monotherapy, descriptive statistic is summarised and median (Q1, Q3) is presented.

## Results

Eighty-six patients enrolled in the trial and participated in the run-in. Mean (SD) total number of prestudy recurrences, including index and qualifying episodes, was 4.7 (1.7). Mean (SD) duration of disease was 2.4 (3.1) years and mean (SD) duration of prior glucocorticoid treatment was 19.9 (36.3) weeks.

Of 86 enrolled patients, 79 were receiving background therapy at baseline. Of these 79 patients, 74 achieved rilonacept monotherapy. The 5 patients not achieving rilonacept monotherapy did not have pericarditis recurrence; discontinuation was due to adverse events (n=3), positive result on tuberculosis testing (n=1), and sponsor decision (n=1).^17^ Of the 79 patients receiving background pericarditis therapy at baseline, 55 (69%) had been receiving double or triple combination treatment for RP at baseline and achieved rilonacept monotherapy during the run-in period. Of these patients, 44% (24/55) were treated with NSAIDs plus colchicine, 24% (13/55) with colchicine plus glucocorticoids, and 33% (18/55) with NSAIDs, colchicine and glucocorticoids. Baseline demographic and clinical characteristics of these patients are shown in table 2.

**Table 2:** Demographic and clinical characteristics of the patients receiving double or triple combination treatment for recurrent pericarditis at baseline

Characteristic	NSAID + colchicine (n=24)	Colchicine + corticosteroid (n=13)	Colchicine + corticosteroid + NSAID (n=18)
Age, years, mean (SD)	44.6 (13.41)	43.5 (16.4)	42.3 (14.7)
Age, years, no. (%)			
12–17	1.0 (4.2)	0	1.0 (5.6)
18–64	22.0 (91.7)	12.0 (92.3)	17.0 (94.4)
65–78	1.0 (4.2)	1.0 (7.7)	0
Female sex, no. (%)	19.0 (79.2)	7.0 (53.8)	11.0 (61.1)
Race*, no. (%)			
White	23.0 (95.8)	12.0 (92.3)	16.0 (88.9)
Black/African American	1.0 (4.2)	0	2.0 (11.1)
Other	0	1.0 (7.7)	0
Aetiology, no. (%)			
Idiopathic	20.0 (83.3)	9.0 (69.2)	15.0 (83.3)
Postpericardiotomy syndrome	3.0 (12.5)	4.0 (30.8)	3.0 (16.7)
Dressler’s syndrome†	1.0 (4.2)	0	0
Duration of prior treatment with corticosteroids, weeks, mean (SD)	0	12.0 (17.7)	11.5 (25.4)
Total episodes, including index and qualifying, no., mean (SD)	4.8 (1.8)	4.2 (0.93)	4.5 (0.99)
Duration of disease, years, mean (SD)	3.6 (4.75)	1.0 (0.4)	1.4 (0.9)
Recurrent episodes per year, mean (SD)	3.8 (5.1)	5.5 (2.9)	5.6 (5.9)
Pain rating (qualifying episode), Numerical Rating Scale Score‡, mean (SD)	6.9 (1.9)	5.1 (1.3)	6.5 (1.7)
Standard C reactive protein (qualifying episode), mg/dL, mean (SD)	5.2 (6.4)	7.5 (7.3)	7.9 (7.4)
Pericarditis manifestations (qualifying episode), no. (%)			
Pericardial effusion§ no. (%)	6.0 (25.0)	7.0 (53.8)	7.0 (38.9)
Pericardial rub, no. (%)	3.0 (12.5)	1.0 (7.7)	5.0 (27.8)
ST elevation or PR depression, no. (%)	3.0 (12.5)	3.0 (23.1)	4.0 (22.2)
Pericarditis treatment at baseline, n (%)			
Non-opioid analgesics	4.0 (16.7)	0	0
Opioid analgesics	4.0 (16.7)	0	0
Aspirin	2.0 (8.3)	0	1.0 (5.6)
Other NSAIDs¶	22.0 (91.7)	0	17.0 (94.4)
Colchicine	24.0 (100)	13.0 (100)	18.0 (100)
Oral corticosteroids	0	13.0 (100)	18.0 (100)
Other	0	1.0 (7.7)	1 (5.6)

*Race was reported by patient.

†The cause of the Dressler syndrome was catheter ablation for atrial fibrillation.

‡Scores on the Numerical Rating Scale for pain range from 0 to 10, with higher scores indicating greater pain severity.

§Pericardial effusion was defined as new or worsening pericardial effusion, independent of the imaging method.

¶Non-steroidal anti-inflammatory drugs.

**Figure 2:**
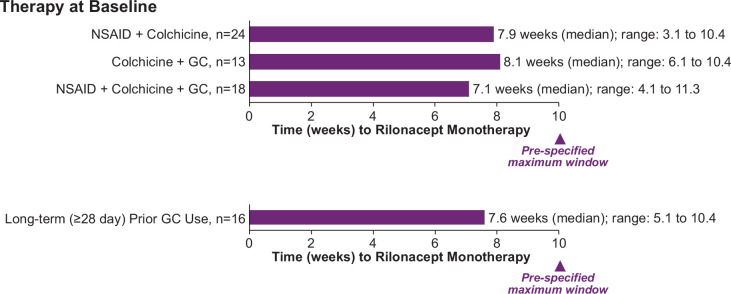
Median time to rilonacept monotherapy, by therapy combination at baseline. GC, glucocorticoid; NSAID, non-steroidal anti-inflammatory drug.

Despite the protocol-specified requirement that patients reach monotherapy rilonacept by week 10 to qualify for randomisation (after 2 weeks of monotherapy), median time to rilonacept monotherapy ranged from 7.1 weeks to 8.1 weeks for patients in the three groups (NSAID plus colchicine and glucocorticoid; colchicine plus glucocorticoid; NSAID plus colchicine; figure 2). Physicians in this study chose to transition their patients to rilonacept monotherapy approximately 2 weeks faster on average than the protocol-stipulated maximum-allowed timeframe to qualify for randomisation. Patients with RP previously treated with long-term (≥28 days) glucocorticoids (n=16) were transitioned by investigators to rilonacept monotherapy in a median of 7.6 weeks (figure 2). Patients treated with ≤15 mg/day (median study dose; n=21) or >median study dose (n=18) glucocorticoids were transitioned to rilonacept monotherapy in a median of 7.3 weeks and 8.0 weeks, respectively.

**Figure 3:**
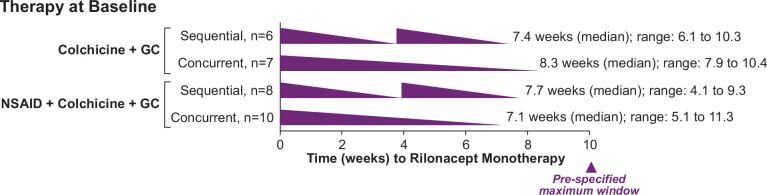
Median time to rilonacept monotherapy in patients receiving treatment with colchicine and glucocorticoids at baseline, by taper approach. GC, glucocorticoid; NSAID, non-steroidal anti-inflammatory drug.

**Figure 4:**
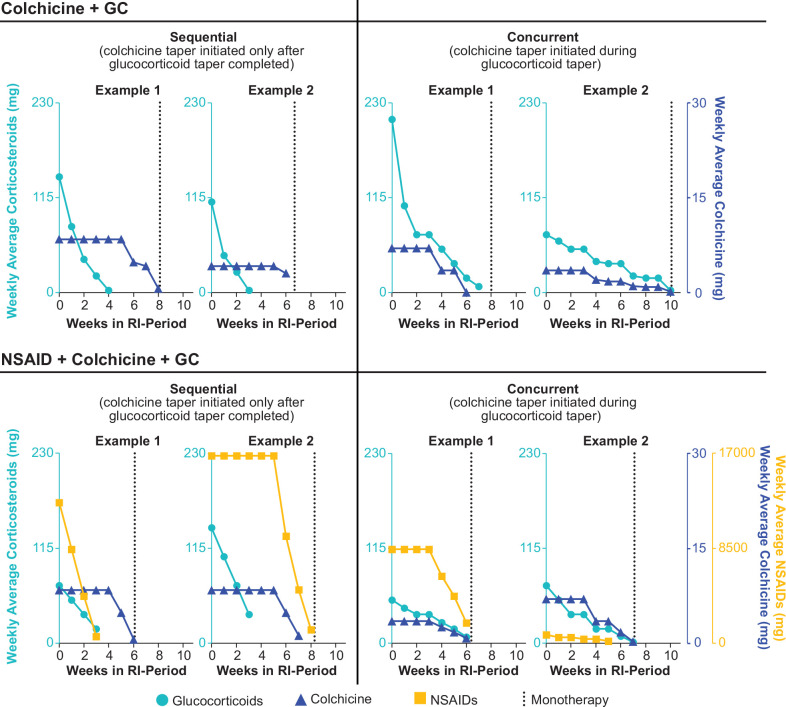
Representative approaches to tapering NSAIDs, colchicine and glucocorticoids. Because these data represent weekly average doses, some data points do not reach zero at the final tapering time point; however, complete taper was achieved. GC, glucocorticoid; NSAID, non-steroidal anti-inflammatory drug; RI, run-in.

Patients receiving both colchicine and glucocorticoids (n=31) were transitioned from background therapies in similar proportions either sequentially (n=14, 45.2%) or concurrently (n=17, 54.8%). Regardless of taper approach, investigators transitioned all patients to rilonacept monotherapy within similar timeframes: sequential (n=14; median, (Q1, Q3), 7.7 (6.1, 8.3) weeks) and concurrent (n=17; median (Q1, Q3), 8.0 (7.1,10.0) weeks) (figure 3). Patients receiving colchicine and glucocorticoids at baseline demonstrated comparable transition time to rilonacept monotherapy (n=24; median (Q1, Q3), 7.9 (5.9, 9.9) weeks). Figure 4 provides examples of representative approaches that investigators took while transitioning patients off standard oral therapies. Of note, there were no pericarditis recurrences during transition to monotherapy.

A trend was observed towards shorter median time to monotherapy in patients with a prestudy annualised incidence of fewer than two episodes per year (median, 6.1 (Q1–Q3, 4.1–8.1) weeks; n=23) than in patients with 2–4 episodes per year (median, 8.3 (Q1–Q3, 7.0–10.0) weeks; n=23) or in those with more than four episodes per year (median, 8.1 (Q1–Q3, 6.6–9.9); n=28). A trend was also observed towards shorter median time to monotherapy in patients with disease duration longer than 2 years (median, 6.1 (Q1–Q3, 4.3–9.3) weeks; n=25) than in patients with disease duration of 1–2 years (median, 8.0 (Q1–Q3, 6.1–9.7) weeks; n=26) or less than 1 year (median, 8.0 (Q1–Q3, 6.7–10.0) weeks; n=23).

## Discussion

Patients entering RHAPSODY were acutely symptomatic despite oral multidrug therapy. Because rilonacept, which is an IL-1α and IL-1β cytokine trap, directly targets the disease mechanism, it had been hypothesised in the trial design that the various standard oral therapies would no longer be needed and could be withdrawn relatively rapidly after rilonacept initiation. This proved to be the case, as these therapies were withdrawn, and rilonacept monotherapy was achieved in a median of 7.9 weeks without pericarditis recurrence.^17^ Rapid resolution of acute episodes shortly after initiation of rilonacept during the run-in period indicates that the targeted IL-1 blockade was both necessary and sufficient to control disease. While it may be more difficult to make conclusions from a post hoc analysis, this analysis revealed the practice patterns clinicians who were initiating rilonacept treatment and transitioning patients off oral therapies took to manage RP medications with the goal of attaining rilonacept monotherapy. Investigators transitioned patients to monotherapy rilonacept over a similar period (just <2 months) in groups of patients receiving combination treatment with commonly used oral therapies for RP (including patients receiving long-term glucocorticoid treatment), regardless of which management approach (sequential or concurrent) was used.

Given the chronicity of RP, clinicians face a conundrum when considering glucocorticoids for disease management: practice guidelines note that chronic use of glucocorticoids is associated with serious side effects, yet the data also show that discontinuation of treatment with glucocorticoids may increase the risk of pericarditis recurrence. Guidelines encourage minimising the risk of recurrence by gradually tapering off glucocorticoids: decreasing the dose by 1–2.5 mg per day every 2–6 weeks over several months when the dose is less than 15 mg/day.^1^ A retrospective claims database study demonstrated that each additional week of glucocorticoid treatment is associated with a 1.11-fold higher risk of related adverse events,^15^ a risk profile suggesting that, if possible, glucocorticoids should be rapidly tapered to reduce such events.

By contrast, use of rilonacept in RHAPSODY allowed for rapid glucocorticoid tapering without precipitating pericarditis recurrence. While clinicians often taper oral therapies taken to manage RP slowly, per available guidelines, those guidelines do not take into consideration a highly targeted, single-agent treatment such as rilonacept. In RHAPSODY, ceasing oral therapies at a rate faster than is usual in clinical practice was possible because of the overlapping and targeted disease coverage provided by rilonacept. Specifically, in patients who had been receiving glucocorticoids in combination with other treatments before study entry—including patients on long-term glucocorticoid therapy—investigators withdrew background treatments after rilonacept initiation (an overlapping approach) on the way to rilonacept monotherapy, which was achieved in a median of 7.1–8.1 weeks; the investigators chose concurrent more often than sequential tapering. This clinical trial observation, taken together with previous findings from studies using anakinra,^21–23^ provides evidence that could support a clinical practice management strategy of more rapid cessation of oral therapies when IL-1 blockade is used to manage RP, potentially minimising adverse events associated with prolonged glucocorticoid exposure. In addition, the safety profile of rilonacept^17^ and once-weekly administration provide additional advantages to transitioning to rilonacept.

RHAPSODY is the first IL-1 antagonist clinical trial in pericarditis in which all patients were treated with monotherapy (the Anakinra Treatment of Recurrent Idiopathic Pericarditis (AIRTRIP) tudy did not require discontinuation of colchicine treatment during the randomised-withdrawal period).^23^ Interestingly, the RHAPSODY investigators made the transition to rilonacept monotherapy in less than the maximum time stipulated by the protocol. In this study, rilonacept initiation in patients who had been experiencing pericarditis recurrence despite oral therapies resulted in rapid resolution of pericarditis episodes (average of 5 days to treatment response) during the run-in period.^17^ This observation may have increased investigator confidence to discontinue oral therapies more rapidly than they might have in their usual clinical practice without the benefit of rilonacept coverage. Although the protocol provided general guidelines for drug management, the investigators were free to use their clinical judgement while discontinuing oral therapies. Therefore, results of this post hoc analysis may more closely mirror real-world clinical tapering practice than would have been expected from a typical clinical trial. Thus, these findings can inform clinical practice and future studies of rilonacept in patients with RP. The ongoing long-term extension of RHAPSODY is designed to provide additional information regarding duration of rilonacept use and possible strategies for cessation of RP pharmacotherapy.

Limitations of this post hoc analysis of RHAPSODY trial data include the small patient population analysed, clinician preference, lack of run-in period events (difficult to show differences), lack of long-term follow-up, and discontinued participation of some patients during the run-in period.

## Conclusion

In this post hoc analysis of RHAPSODY data, investigators initiating rilonacept treatment in patients with RP rapidly transitioned the patients from oral multidrug therapy (including long-term glucocorticoid treatment) to rilonacept monotherapy. Patients experienced rapid treatment response to rilonacept therapy and discontinued treatment with NSAIDs, colchicine, and/or glucocorticoids in a median of 7.1–8.1 weeks without recurrence of pericarditis, regardless of whether a sequential or concurrent tapering approach was employed. This analysis provides additional insight for clinicians considering even more rapid cessation of oral therapies when incorporating rilonacept into the treatment paradigm for patients with RP.

## Data Availability

Data are available in a public, open access repository. Data are available upon reasonable request. De-identified participant data will be available, one year after study completion, upon reasonable written request to researchers whose proposed use of the data has been approved. Requests may be directed to Dr John F Paolini, ORCID ID: 0000-0001-7622-8851. Data will not be provided to requesters with potential or actual conflicts of interest, including individuals requesting access for commercial, competitive or legal purposes. Data access may be precluded for studies in which clinical data were collected subject to legal, contractual or consent provisions that prohibit transfer to third parties. All those receiving access to data will be required to enter into a Data Use Agreement (DUA), which shall contain terms and conditions that are customary for similar agreements and similar companies in the industry. Data available to the public including the study protocol can be found at: (data set) Klein AL, Imazio M, Cremer P, *et al*. Phase 3 trial of interleukin-1 trap rilonacept in recurrent pericarditis. N Engl J Med 2021;384:31-41. DOI: 10.1056/NEJMoa2027892.

## References

[1] Adler Y , Charron P , Imazio M , et al . 2015 ESC Guidelines for the diagnosis and management of pericardial diseases: The Task Force for the Diagnosis and Management of Pericardial Diseases of the European Society of Cardiology (ESC)Endorsed by: The European Association for Cardio-Thoracic Surgery (EACTS). Eur Heart J 2015;36:2921–64. 10.1093/eurheartj/ehv318 26320112 PMC7539677

[2] Vecchié A , Chiabrando JG , Dell MS , et al . Clinical presentation and outcomes of acute pericarditis in a large urban hospital in the United States of America. Chest 2020;158:2556–67. 10.1016/j.chest.2020.07.039 32717264 PMC7768931

[3] Bizzi E , Picchi C , Mastrangelo G , et al . Recent advances in pericarditis. Eur J Intern Med 2022;95:24–31. 10.1016/j.ejim.2021.09.002 34556390

[4] Imazio M , Brucato A , Cemin R , et al . A randomized trial of colchicine for acute pericarditis. N Engl J Med 2013;369:1522–8. 10.1056/NEJMoa1208536 23992557

[5] Imazio M , Bobbio M , Cecchi E , et al . Colchicine in addition to conventional therapy for acute pericarditis: results of the colchicine for acute pericarditis (cope) trial. Circulation 2005;112:2012–6. 10.1161/CIRCULATIONAHA.105.542738 16186437

[6] Hernández-Díaz S , Rodríguez LA . Association between nonsteroidal anti-inflammatory drugs and upper gastrointestinal tract bleeding/perforation: an overview of epidemiologic studies published in the 1990s. Arch Intern Med 2000;160:2093–9. 10.1001/archinte.160.14.2093 10904451

[7] Imazio M , Gaita F , LeWinter M . Evaluation and treatment of pericarditis: a systematic review. JAMA 2015;314:1498–506. 10.1001/jama.2015.12763 26461998

[8] Chiabrando JG , Bonaventura A , Vecchié A , et al . Management of Acute and Recurrent Pericarditis: JACC State-of-the-Art Review. J Am Coll Cardiol 2020;75:76–92. 10.1016/j.jacc.2019.11.021 31918837

[9] Imazio M , Brucato A , Cumetti D , et al . Corticosteroids for recurrent pericarditis: high versus low doses: a nonrandomized observation. Circulation 2008;118:667–71. 10.1161/CIRCULATIONAHA.107.761064 18645054

[10] Rice JB , White AG , Scarpati LM , et al . Long-term systemic corticosteroid exposure: a systematic literature review. Clin Ther 2017;39:2216–29. 10.1016/j.clinthera.2017.09.011 29055500

[11] Broersen LHA , Pereira AM , Jørgensen JOL , et al . Adrenal insufficiency in corticosteroids use: systematic review and meta-analysis. J Clin Endocrinol Metab 2015;100:2171–80. 10.1210/jc.2015-1218 25844620

[12] Bornstein SR , Allolio B , Arlt W , et al . Diagnosis and treatment of primary adrenal insufficiency: an endocrine Society clinical practice guideline. J Clin Endocrinol Metab 2016;101:364–89. 10.1210/jc.2015-1710 26760044 PMC4880116

[13] Imazio M , Bobbio M , Cecchi E , et al . Colchicine as first-choice therapy for recurrent pericarditis: results of the core (colchicine for recurrent pericarditis) trial. Arch Intern Med 2005;165:1987–91. 10.1001/archinte.165.17.1987 16186468

[14] Schwier NC , Luis SA , Hu X , et al . PCV5 risk factors associated with recurrence and Corticosteroid-Associated adverse events in patients with recurrent pericarditis. Value Health 2021;24:S67. 10.1016/j.jval.2021.04.345

[15] Schwier NC , Luis SA , Hu X , et al . Risk factors associated with recurrence and corticosteroid-associated adverse events in patients with recurrent pericarditis [poster]. Presented at: Annual Meeting of the International Society for Pharmacoeconomics and Outcomes Research; May 17-20, 2021.

[16] Dinarello CA , Simon A , van der Meer JWM . Treating inflammation by blocking interleukin-1 in a broad spectrum of diseases. Nat Rev Drug Discov 2012;11:633–52. 10.1038/nrd3800 22850787 PMC3644509

[17] Klein AL , Imazio M , Cremer P , et al . Phase 3 trial of interleukin-1 trap rilonacept in recurrent pericarditis. N Engl J Med 2021;384:31–41. 10.1056/NEJMoa2027892 33200890

[18] Regeneron Pharmaceuticals . Arcalyst [package insert]. Tarrytown, NY, 2016.

[19] Klein AL , Lin D , Cremer PC , et al . Efficacy and safety of rilonacept for recurrent pericarditis: results from a phase II clinical trial. Heart 2020;107:488–96. 10.1136/heartjnl-2020-317928 33229362 PMC7925818

[20] Klein AL , Imazio M , Brucato A , et al . RHAPSODY: rationale for and design of a pivotal phase 3 trial to assess efficacy and safety of rilonacept, an interleukin-1α and interleukin-1β trap, in patients with recurrent pericarditis. Am Heart J 2020;228:81–90. 10.1016/j.ahj.2020.07.004 32866928

[21] Finetti M , Insalaco A , Cantarini L , et al . Long-term efficacy of interleukin-1 receptor antagonist (anakinra) in corticosteroid-dependent and colchicine-resistant recurrent pericarditis. J Pediatr 2014;164:1425–31. 10.1016/j.jpeds.2014.01.065 24630353

[22] Lazaros G , Vasileiou P , Koutsianas C , et al . Anakinra for the management of resistant idiopathic recurrent pericarditis. initial experience in 10 adult cases. Ann Rheum Dis 2014;73:2215–7. 10.1136/annrheumdis-2014-205990 25165036

[23] Brucato A , Imazio M , Gattorno M , et al . Effect of anakinra on recurrent pericarditis among patients with colchicine resistance and corticosteroid dependence: the AIRTRIP randomized clinical trial. JAMA 2016;316:1906–12. 10.1001/jama.2016.15826 27825009

