# Correction: *Transition to rilonacept monotherapy from oral therapies in patients with recurrent pericarditis*

**DOI:** 10.1136/heartjnl-2022-321328corr1

**Published:** 2023-12-19

Brucato A, Wheeler A, Luis SA, et al. Transition to rilonacept monotherapy from oral therapies in patients with recurrent pericarditis. *Heart* 2023; **109**: 297-304.

This article has been corrected since it was first published. A sentence in the Results section incorrectly stated “patients receiving colchicine and **glucocorticoids** at baseline demonstrated comparable transition to rilonacept monotherapy”. This has now been corrected to “patients receiving colchicine and **NSAIDs** at baseline demonstrated comparable transition to rilonacept monotherapy”.

